# Teaching nature of science in introductory biology: Impacts on students’ acceptance of biological evolution

**DOI:** 10.1371/journal.pone.0289680

**Published:** 2023-08-10

**Authors:** Jeremy D. Sloane, Lindsay B. Wheeler, Jessamyn S. Manson

**Affiliations:** 1 Biology Department, Skidmore College, Saratoga Springs, New York, United States of America; 2 Center for Teaching Excellence, University of Virginia, Charlottesville, Virginia, United States of America; 3 Department of Biology, University of Virginia, Charlottesville, Virginia, United States of America; University of Padova, ITALY

## Abstract

The present study investigates the impact of explicit, reflective Nature of Science instruction on students’ evolution acceptance, understanding of evolution as a theory, and understanding of Nature of Science in an introductory biology course. Results revealed similar improvement in evolution acceptance in both the treatment and control groups, but also that Nature of Science instruction had disproportionately large impacts on evolution acceptance for women and individuals who already had high acceptance. We also found evidence of relationships between understanding and acceptance of evolution and Nature of Science understanding, particularly the creativity aspect of Nature of Science. Together, these results suggest that targeted Nature of Science instruction can have differential impacts on students with particular characteristics, such as women and individuals with high acceptance, but also point to the need to consider additional interventions that can reach men and individuals with low acceptance.

## Introduction

Despite the importance of evolution in explaining the diversity of life on Earth, a troublingly high percentage of the American public continues to doubt the veracity of evolutionary biology. Understanding of the Nature of Science (NOS) has been found to be a significant predictor of evolution acceptance, but studies exploring the impact of NOS instruction on evolution acceptance have thus far been limited in number as well as in scope and nature. The present study investigates the impact of explicit, reflective NOS instruction on evolution acceptance, understanding of evolution as a theory, and understanding of NOS in post-secondary students enrolled in an introductory biology course.

### Literature review

#### Importance of evolution

Evolutionary biologist Theodosius Dobzhansky once stated, “Nothing in biology makes sense except in the light of evolution” [1]. Indeed, there is little scientific doubt regarding the veracity of evolutionary theory and its ability to explain the diversity of life on Earth, and this view has been articulated by all major scientific associations. The National Academy of Sciences (NAS) states, “…there is no debate within the scientific community over whether evolution occurred, and there is no evidence that evolution did not occur” [2], while the American Association for the Advancement of Science (AAAS) calls evolution “one of the most robust products of scientific inquiry” and “the foundation for research in many areas of biology” [3]. The American Institute of Biological Sciences singles out evolution as the only viable theory for explaining the diversity of life forms, calling it “the only scientifically defensible explanation for the origin of life and development of species” [4]. The theory of evolution provides the fundamental framework for making predictions about changes in life over time. To study biology without accepting evolution limits one’s ability to understand the mechanisms, patterns and processes that govern life on earth.

Despite near-unanimous consensus regarding evolution’s status as the predominant theory in explaining the origin and diversity of life, no such consensus exists among members of the general public. According to the Pew Research Center [5], 18% or 31% of participants polled about evolution and God rejected evolution as a scientific theory explaining diversity of life forms (The percentage of participants who reject evolution varies based on how the question is asked.). Suffice to say, although there is no consensus regarding the percentage of the public that rejects evolution, that number is certainly higher than the 2% of AAAS scientists who reject it [6]. The disparity between scientific and the general public’s acceptance of evolution is troubling, as the theory of evolution is critical for understanding the world around us, particularly the pressing concerns of the COVID-19 pandemic, the role that evolution plays in the transmission of diseases between species and the appearance of new disease variants. Many other societal challenges, such as species responses to climate change, antibiotic and pesticide resistance and the consequences of contemporary agriculture, specifically genetic modification and monocultural crop production, are all most comprehensively understood through the lens of evolution. For many years, researchers have investigated factors that influence acceptance of evolution in order to provide educators guidance to help improve acceptance among members, or soon-to-be members, of the voting general public.

#### Factors influencing acceptance of evolution

Within the context of higher education, there are a few studies that identify factors that influence students’ acceptance of evolution. These factors include religiosity (i.e., the extent to which religious beliefs influence decision making), understanding of Nature of Science (NOS) (defined below), and understanding of evolution (evolutionary content knowledge). For example, Glaze and colleagues investigated the significant predictors of evolution acceptance among preservice science teachers [7]. Their final model explained 45% of variance in evolution acceptance with four variables, which were: participants’ view of the influence of their religious beliefs on decision-making related to science, understanding of Nature of Science (NOS), evolution content knowledge, and STEM influences outside the classroom (listed in order from most explanatory to least explanatory). Similarly, Dunk and colleagues [8] assessed the factors which predicted evolution acceptance among students enrolled in an Introduction to Anatomy and Physiology course. The results from this study differed somewhat from those of Glaze and colleagues [7]; the most significant predictor of evolution acceptance was understanding of NOS, followed by religiosity, epistemological sophistication, and evolutionary content knowledge. Lombrozo and colleagues [9] quantified correlations between evolution acceptance and several other factors among university undergraduates and found roughly equal correlations between evolution acceptance and religiosity, NOS understanding, and attitudes towards science.

In a study of Korean pre-service teachers, Kim and Nehm [10] explored relationships between participants’ acceptance of evolution, understanding of evolution, and understanding of NOS. The authors found significant positive relationships between NOS understanding and acceptance of evolution. Further, there were significant differences between men and women in the study; men had significantly higher evolutionary acceptance and understanding of evolution than woman. This is an intriguing finding as prior research has not found gender to be a significant predictor of evolution acceptance [7,8]. No differences existed in NOS understanding between men and women. The authors also found differences in NOS understanding based on religion. Partin and colleagues [11] similarly studied the relationships between NOS understanding, acceptance of evolution, and understanding of evolution for undergraduate biology students. The authors identified that both understanding and acceptance of evolution were significant predictors for students’ NOS understanding and, along with parental education and religiosity, accounted for 24% of variance in the model.

In combination, what these studies suggests is that NOS understanding, understanding of evolution, religiosity, and demographics (i.e., gender, parental education) play important roles in students’ acceptance of evolution. Unlike demographics and religion, students’ understanding of NOS and understanding of evolution can shift based on instruction [12]. However, these two types of knowledge differ; whereas evolution is a concept embedded within a discipline, NOS is a cross-disciplinary understanding. While research demonstrates a variety of ‘active learning’ strategies promote content knowledge gains [13,14], research suggests that a more nuanced and specific approach to NOS instruction is needed to improve NOS understanding [15,16]. Below we provide a conceptual framework for understanding NOS and the characteristics of effective NOS instruction that situate the present study.

### Conceptual framework

#### Nature of science characteristics

Nature of Science encompasses the values and beliefs inherent to the development of scientific knowledge and includes a number of tenets that characterize these values [17]. While there is no consensus on these NOS characteristics, we used the following tenets as the foundation for our participants’ NOS understandings: scientific knowledge is empirical, reliable yet tentative, based on observation and inference, the product of creative thinking, subjective and theory-laden, and socially and culturally embedded [18,19]. Two additional tenets include: scientific theories and laws are different types of scientific knowledge and there are multiple methods for gaining knowledge in science (e.g., experimental, observational) [18,19].

#### Nature of science instruction

It is generally agreed that an explicit, reflective approach to teaching NOS is most effective for improving students’ understanding of NOS [12,20,21]. In this approach, NOS is taught through purposeful integration of NOS tenets into the course. It is not enough for students to experience science [22]; students must also learn about what it means to ‘do’ science and what it takes to gain scientific knowledge. This NOS integration requires an instructor to consider the context in which the instruction occurs, and researchers have demonstrated the value in embedding explicit, reflective NOS instruction across a context continuum [12,16]. In minimally contextualized NOS instruction students may engage in a non-content based exploration or activity that illustrates a particular NOS tenet. The NOS tenet is then connected to the course content at the end of the activity. Conversely, in highly contextualized NOS instruction the course content serves as the basis for understanding a NOS tenet. We used a cross-context, explicit, reflective approach to NOS instruction in the present study and detail our interventions in the methods below.

While much of the NOS research focuses on K-12 instruction [23,24] there are a number of studies exploring the impact of NOS instruction on undergraduate students’ NOS understandings [11,15]. Of these studies, only a few focus on NOS instruction in undergraduate biology contexts. For example, in a study of introductory biology courses, Schussler and colleagues [15] explored the impact of both inquiry and NOS instruction on students’ NOS understandings. The authors found students’ NOS understandings improved the most in non-inquiry contexts where NOS instruction was explicit and reflective and conclude that ‘doing’ science (i.e., inquiry) may preclude ‘understanding’ science (i.e., NOS). They suggest that embedding NOS instruction into lecture is one alternative approach for supporting students’ understanding of NOS.

Other biology education literature suggests that NOS instruction embedded within an evolutionary context may be effective in supporting students’ understanding of NOS [11,25,26].

Nelson and colleagues identify six key factors influencing the instructional success of evolution education based on a review of the extant literature [25]. They include: (1) foster a deep understanding of NOS; (2) use NOS as a lens for evolution instruction; (3) explicitly compare evolution to alternative explanations; (4) focus on human evolution (where possible); (5) explicitly recognize the power of historical inference and (6) use active, social learning. Regarding effective NOS within the context of evolutionary biology, Scharmann [21] provides four insights: (1) teach explicit NOS principles first; (2) integrate evolution as a theme throughout a course in introductory biology (but after NOS principles have been introduced); (3) use active learning pedagogies; and (4) use non-threatening alternative assessments to enhance student learning and acceptance of evolutionary science. Both of these sources cite the importance of NOS instruction contextualized within evolution instruction as well as active learning strategies to enhance understanding.

Only one study, to our knowledge, empirically explored how NOS instruction influences acceptance of evolution. In a study of high school biology students in Chile, Cofre and colleagues [23] investigated whether NOS instruction influenced students’ evolution acceptance. The authors found that students’ acceptance of evolution improved for those in the treatment group (i.e., received NOS instruction), while it did not improve for the control group (i.e., no NOS instruction). While promising findings, further exploration into the relationships between NOS instruction and acceptance of evolution are needed, particularly in a higher education context.

#### Purpose

Despite the importance of NOS understanding to students’ acceptance of evolution, there is little research exploring how these concepts relate to each other within the context of NOS instruction in undergraduate biology courses. Most studies exploring NOS instruction and acceptance of evolution also do not employ treatment and control groups, and we are also unaware of any prior work investigating how NOS instruction influences evolution acceptance for individuals with different genders or levels of acceptance. Thus, the purpose of our study was to investigate the potential influence of NOS instruction on evolution acceptance, understanding of evolution as a theory, and NOS understanding among students enrolled in a large-enrolment introductory biology course. The research questions which guided our study were:

1. *What shifts*, *if any*, *occurred in students’ acceptance of evolution after exposure to explicit*, *reflective instruction on NOS*, *and what differences existed in evolution acceptance between students who receive the NOS instruction and those who did not*? *Did this change based on students’ gender*?
2. *What shifts*, *if any*, *occurred in NOS understanding after explicit*, *reflective instruction on NOS*, *and what differences existed in NOS understanding between students who received the NOS instruction and those who did not*?
3. *To what extent did understanding of evolution as a scientific theory differ between students who did and did not receive explicit*, *reflective instruction on NOS*?
4. *What relationships existed between acceptance of evolution*, *understanding of NOS*, *and understanding of evolution as a scientific theory for those who did and did not engage with NOS instruction*? *How did these relationships differ based on gender and prior evolution acceptance levels for each group*?

We hypothesized that NOS instruction would improve acceptance of evolution, and that participants who received NOS instruction would have higher levels of acceptance than those who did not. We also hypothesized that NOS instruction would improve students understanding of both NOS and evolution as a theory, and that students who received this instruction would have higher levels of NOS understanding and understanding of evolution as a theory than those who did not.

## Methods

This convergent mixed methods study [27] with a quantitative emphasis took place in an introductory biology course at a mid-size research intensive public university in the mid-Atlantic United States during the Spring 2019 semester. Participants included students in two sections of the course taught by the same instructor, Researcher C. One section of the course was designated as the *Treatment* group (BIO 220-T), who received a NOS intervention, and the other section was the *Control* group (BIO 220-C), who received no NOS intervention. This study was approved by the University of Virginia’s Institutional Review Board for the Social and Behavioral Sciences (IRB-SBS), and the researchers obtained written consent from students to voluntarily participate in the research study (IRB# 2018-0514-00). Details about the course, intervention, participants, and data are below.

### Context

The course that served as the context for the study was a 4-credit, second semester introductory biology course (BIO 220). The course is one of two introductory biology courses required for all biology majors and there are no prerequisite courses required for enrolment. The course had two large lecture sections (>400 students per section) that met three times a week for 50-minutes. The course also included a required laboratory component run by TAs in small sections of ~24 students that met 2.5 hours per week. Students selected both their lecture and laboratory times when registering for the course.

The course focused on organismal and evolutionary biology, using evolution as the framework to teach about organismal diversity, as well as vertebrate form and function. The first three weeks of the course were specifically designed to give students a strong foundation in the tenets of natural selection, the mechanisms of and evidence for evolution, how evolution leads to speciation and subsequently explains patterns of biological diversity (See S1 Appendix for course syllabus). During class time, the instructor lectured using PowerPoint slides and included iClicker questions for students to discuss and respond to throughout each class period. Class sessions were complemented by online homework and associated lab activities that provided students with opportunities to apply concepts from class.

**Fig 1:**
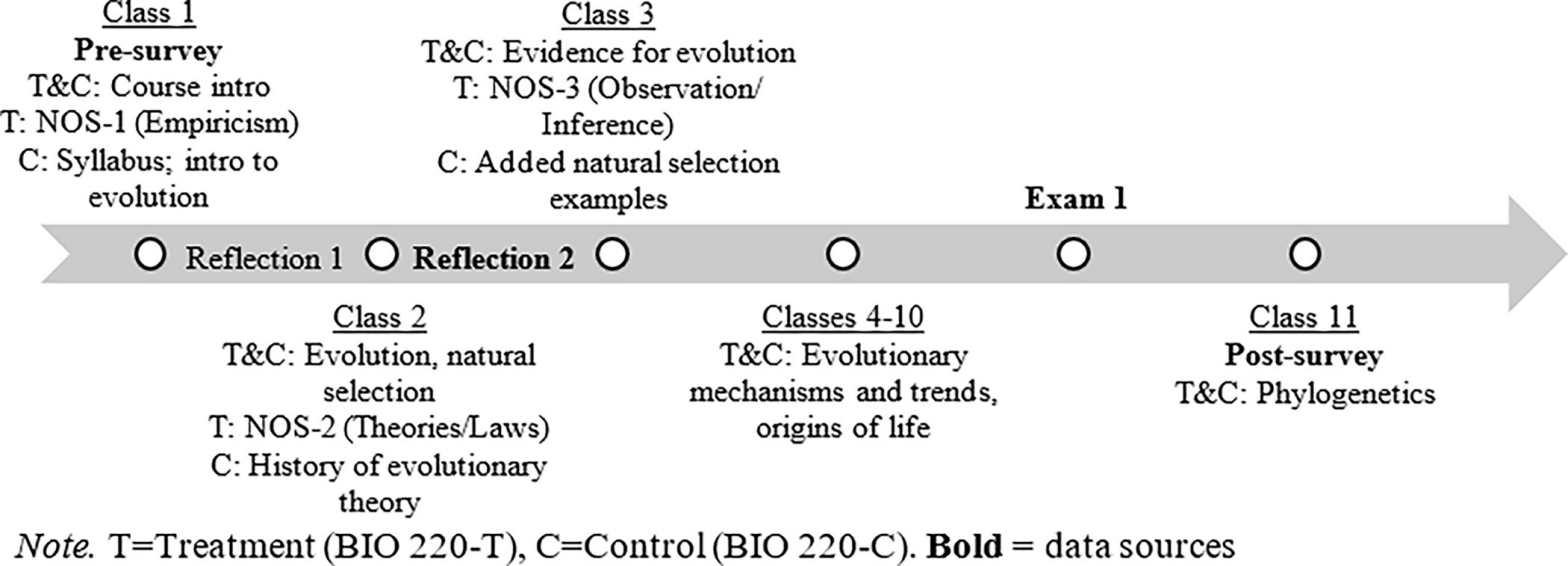
Overview of BIO 220 course timeline.

The study occurred in the first four weeks of classes during the unit on evolution. In both sections, BIO 220-T and BIO 220-C, the instructor covered similar material, as described in the syllabus. The NOS intervention occurred in BIO 220-T during the first three class periods (60 minutes total) and aligned with best practices in NOS instruction [15,16]. Two of the NOS activities were taught by Researcher B and focused on one main NOS tenet at a time (details about the instructional methods can be found in S2 Appendix). The activities included explicit NOS instruction, opportunities for students to engage with and reflect on each of the tenets, and incorporated activities along a context continuum. In lieu of the NOS activities, students in BIO 220-C received further lecture on the day’s topic from the instructor. See Fig 1 for overview of the study.

### Participants

Students enrolled in BIO 220 were recruited to participate in this IRB-approved study. Of the 840 total students in the course, 627 (75.4%) voluntarily consented to the study via written consent and completed both the pre and post surveys. This group of participants was used to answer research questions 1 and 2. The demographic breakdowns of these participants were similar across BIO 220-T and BIO 220-C and similar to the overall demographics of the students enrolled in their respective section (Table 1). A total of 308 of the 627 participants (49.1%) consented to the use of their reflection assignment and exam 1 answers, which were used to answer research questions 3 and 4.

**Table 1:** Overview of student demographics in treatment (BIO-220-T) and control (BIO-220-C) sections.

Demographics		BIO 220-T,		BIO 220-C,	
		Participants, n = 314	All students, n = 419	Participants, n = 313 (%)	All students, n = 421
Gender	Women	238 (75.8)	305 (72.8)	229 (73.2)	299 (71.0)
	Men	75 (23.9)	114 (27.2)	82 (26.2)	122 (29.0)
	Unreported	1 (.3)	0 (0)	2 (.6)	0 (0)
Race	Caucasian	187 (59.6)	242 (57.8)	188 (60.1)	215 (51.1)
	Asian	69 (22.0)	74 (17.7)	66 (21.1)	88 (20.9)
	African-American	25 (8.0)	37 (8.8)	20 (6.4)	34 (8.1)
	Multi-racial	21 (6.7)	24 (5.7)	24 (7.7)	29 (6.9)
	Other/unreported	8 (2.5)	21 (5.0)	7 (2.2)	28 (6.6)
	Hispanic	4 (1.3)	23 (5.5)	7 (2.2)	25 (5.9)
	Pacific-island	0 (0)	0 (0)	1 (.3)	2 (.4)
Pre-health major		237 (75.5)	NR	245 (78.3)	NR
In-state student		220 (70.1)	299 (71.4)	225 (71.9)	313 (74.3)
First-generation college student		41 (13.1)	52 (12.4)	48 (15.3)	54 (12.8)
International student		7 (2.2)	6 (1.4)	9 (2.9)	6 (1.4)

*Notes*. NR = not reported. We did not obtain demographic information for participants used to answer research questions 3 & 4.

### Data collection

Quantitative data sources included participants’ pre and post survey responses as well as exam responses. The pre/post survey contained 20 Likert questions from the Measure of Acceptance of the Theory of Evolution (MATE) [28] and 21 Likert questions from the Student Understanding of Science and Scientific Inquiry (SUSSI) [29]. The post-survey included additional demographic questions. The survey was administered through Qualtrics and completed during class time. Students took the pre-survey on the first day of class and the post-survey in the first class following exam 1 (class 11). Students’ responses on an evolution and NOS-related multiple choice exam question were also collected. This question was:

Evolution is a scientific theory because:

a) more evidence is needed before it will be accepted by the scientific community.
b) it continues to be revised, which prevents the development of a fixed evolutionary law.
c) it is a process that can be driven by many different mechanisms.
*d) it provides a comprehensive explanation for natural patterns within populations and across species.

* = correct answer

Students’ reflection assignment responses served as the qualitative data source. The assignment was part of the course homework requirements and were submitted in the Learning Management System (LMS) following class 2. The reflection prompt, which was the same for students in both groups, asked students to respond to the question ‘Is evolution just a theory? Explain your reasoning’.

### Data analysis

For the quantitative data (i.e., surveys, exam responses), we first reverse coded MATE and SUSSI Likert questions (see S3 Appendix for details). For the MATE questions, we calculated Cronbach’s alpha to confirm the questions were reliable (n = 20, α = .938), then created a sum MATE score (out of 100) for each participants’ pre- and post- survey. Because we were interested in how students of varying degrees of evolution acceptance would respond to the intervention, we also categorized participants’ pre-MATE scores into low (<65), moderate (65–75), and high acceptance (>76). Because the SUSSI has not been previously validated, we conducted an Exploratory Factor Analysis (EFA) to identify the possible NOS factors. Based on best practices in EFA [30], we used principle axis factoring with a promax rotation, which resulted in a final model with four factors explaining 35% of the variance (Table 2). Due to the limited sample size of our quantitative data (n = 627), we were unable to randomly split the data to run both EFA and confirmatory factor analysis (CFA). A mean value was then created for the four factors for each participants’ pre- and post- survey. Participants’ multiple choice NOS exam question was coded as correct (1) or incorrect (0).

**Table 2:** Factor loadings for SUSSI questions.

Instrument item	Factor			
	1	2	3	4
*Factor 1*: *Science is Dynamic*				
Scientific theories may be completely replaced by new theories in light of new evidence.	.729			
Scientific theories are subject to on-going testing and revision.	.639			
Scientific theories may be changed because scientists reinterpret existing observations	.617			
Scientists may make different interpretations based on the same observations.	.614			
Scientists use a variety of methods to produce fruitful results	.587			
Experiments are not the only means used in the development of scientific knowledge.	.510			
*Factor 2*: *Scientists use creativity*				
Scientists do not use their imagination and creativity because these conflict with their logical reasoning. (reverse coded)		.830		
Scientists do not use their imagination and creativity because these can interfere with objectivity. (reverse)		.779		
Scientists use their imagination and creativity when they analyze and interpret data.		.473		
*Factor 3*: *Science is culturally embedded*				
Cultural values and expectations determine how science is conducted and accepted.			.969	
Cultural values and expectations determine what science is conducted and accepted.			.639	
*Factor 4*: *Science is subjective*				
When scientists use the scientific method correctly, their results are true and accurate. (reverse)				.503
Scientists’ observations of the same event will be the same because observations are facts. (reverse)				.479
Scientists follow the same step-by-step scientific method. (reverse)				.439
Factor pre-survey Mean (SD)	4.29 (.42)	3.55 (.74)	3.70 (.81)	3.29 (.68)
Eigenvalue	3.367	1.509	1.258	.815
Percentage of variance	16.84%	7.55%	6.29%	4.07%
Construct reliability	.780	.722	.748	.442

*Note*. Eight SUSSI questions did not load on to these factors and were excluded from further analysis.

For the qualitative data (i.e., reflection assignment responses), we used a constant comparative approach [31]. First, Researcher A inductively coded a random sample of 25 responses from each group (50 total) to develop a coding scheme. Researcher C then used the coding scheme to independently code the same responses, and the two researchers met to discuss and finalize the coding scheme. This coding scheme (Table 3) was then used by Researchers A and C to independently code a randomly selected, blinded subset (n = 104, 25%) of the data, meaning the researchers did not know whether the responses were from students in BIO 220-T or BIO 220-C. Inter-rater reliability was 94.7%, and all coding discrepancies were resolved upon discussion. The remaining blinded qualitative responses (n = 294) were split and coded by either Researcher A or C.

**Table 3:** Overview of qualitative coding scheme for participant reflective assignment.

Code	The student indicated that…	Example quote
*Aligned*		
Fact	evolution is a fact	“Evolution is a fact, because we know that it happens.”
Explains	evolution is a theory because it has explanatory power	“Evolution is a theory, as it explains the phenomenon of species changing over time.”
Evidence	evolution is supported by evidence	“Evolution is a theory…that is supported by a large body of evidence.”
Refined	evolutionary theory has been refined over time (or can be refined)	“Evolution is a theory that is changing over time as more evidence and ideas are being discovered.”
Difference	there is a difference between the scientific definition of a theory and the colloquial/everyday definition of theory	“Evolution is a theory in the scientific sense, but not the way we think of theory in every day terminology.”
Tested	evolutionary theory has been tested	“Evolution is a theory because it has been tested multiple times through observation and experimentation.”
Predict	evolutionary theory allows scientists to make predictions	“The theory of evolution…provide[s] a basis for making predictions about biological phenomena.”
*Misaligned*		
Proven	something can be “proven” or “disproven”	“Evolution is a theory that is based on many hypothesis [sic] that have been proven.” “Since being described during the 1800s, no new scientific evidence has surfaced to disprove [evolution], nor even attempted to disprove it.”
Describe	evolution has descriptive power (as such, student may refer to it as a law)	“[Evolution] can also be viewed as a law because it describes the way in which something happens.”

The coding of the entire qualitative data set (n = 398) was then quantified (i.e., presence of code in response = 1) and summed to create two scores representing participants *understanding of evolution*; an ‘aligned’ and ‘misaligned’ score. For example, if a participant’s reflective assignment response contained comments coded for ‘fact’, ‘refined’, and ‘proven’, then they would receive a score of 2 for *aligned understanding of evolutionary theory* (for the terms ‘fact’ and ‘refined’) and a score of 1 for *misaligned understanding of evolutionary theory* (for the term ‘proven’).

To answer research question 1, we were unable to run a 2 (pre/post) x 2 (treatment vs. control) mixed model ANOVA due to violations of normality (i.e., non-normally distributed Q-Q plots of studentized residuals for both pre and post MATE scores) and homogeneity of variance (i.e., significant Levene’s test, ps < .05). Therefore, we ran paired t-tests for both BIO 220-T and BIO 220-C to identify changes in pre/post acceptance of evolution. We also calculated a MATE change score (MATE_post_−MATE_pre_) and ran independent t-tests for each group (i.e., BIO 220-T and BIO 220-C) to identify differences in these changes based on different demographic characteristics such as gender and prior evolution acceptance levels. We also used independent t-tests to identify differences in post-MATE scores between BIO 220-T and BIO 220-C based on gender and prior acceptance levels. Finally, we calculated effect sizes of our analyses, reported as Cohen’s d.

Similarly, to answer research question 2, we were unable to run a 2 (pre/post) x 2 (treatment vs. control) x 4 (SUSSI factors) mixed model ANOVA due to violations of homogeneity of variance (i.e., significant Levene’s test, ps < .05) and homogeneity of covariance (i.e., significant Box’s test of p < 0.05). Therefore, we ran paired sample t-tests to identify pre-post differences in the four SUSSI factor scores for both groups. Independent t-tests were also utilized to test for differences between BIO 220-T and BIO 220-C regarding each pre and post scores for each SUSSI factor. Adjusted p-values were used to account for multiple tests (p < 0.05/4 = .0125).

To answer research question 3, we ran chi-square tests of independence to identify associations between participants’ individual reflection code scores (i.e., presence or absence of each code) and group (i.e., BIO 220-T and BIO 220-C). Because we did not include demographic questions with consent to use the reflection data, we were unable to disaggregate these findings by gender. However, we did conduct a Mann-Whitney U non-parametrics test to identify differences in treatment and control participants’ aligned (range 0–7) and mis-aligned (range 0–2) summed scores as a proxy for understanding of evolution as a theory. We ran a chi-square test of independence to identify differences in treatment and control participants’ exam question response as an additional measure of participants’ understanding of evolution as a theory.

To answer research question 4, we ran a Pearson correlation analysis to identify significant relationships between acceptance of evolution (i.e., pre- and post-MATE scores) and understanding of NOS factors (i.e., science is dynamic, scientists use creativity, science is culturally embedded, science is subjective) for both groups. We also identified significant differences between the strength of MATE/NOS relationships between BIO-220-T and BIO-220-C. Because of the non-continuous nature of participants’ understanding of evolution exam question response, we ran a Spearman’s Rho correlational analysis to identify significant relationships between understanding of evolution, acceptance of evolution, and understanding of NOS for both groups. Using a NOS change score for each of the four factors (e.g., NOS_creative(post)_−NOS_creative(pre)_, we also ran an ANOVA to identify differences in NOS understanding for participants with low/moderate acceptance of evolution (i.e., pre-MATE <75) compared to participants with high incoming acceptance of evolution (i.e., pre-MATE >76). In other words, we sought to understand whether there were differences in improving NOS understanding for participants with different levels of incoming acceptance of evolution. Based on these analyses, we ran an additional ANOVA only for participants with high incoming acceptance of evolution (i.e., pre-MATE >76) to identify differences between NOS change scores for each of the four factors for men and women participants.

## Results

RQ1: *What shifts*, *if any*, *occurred in students’ acceptance of evolution after exposure to explicit*, *reflective instruction on NOS*, *and what differences existed in evolution acceptance between students who received the NOS instruction and those who did not*?

**Fig 2:**
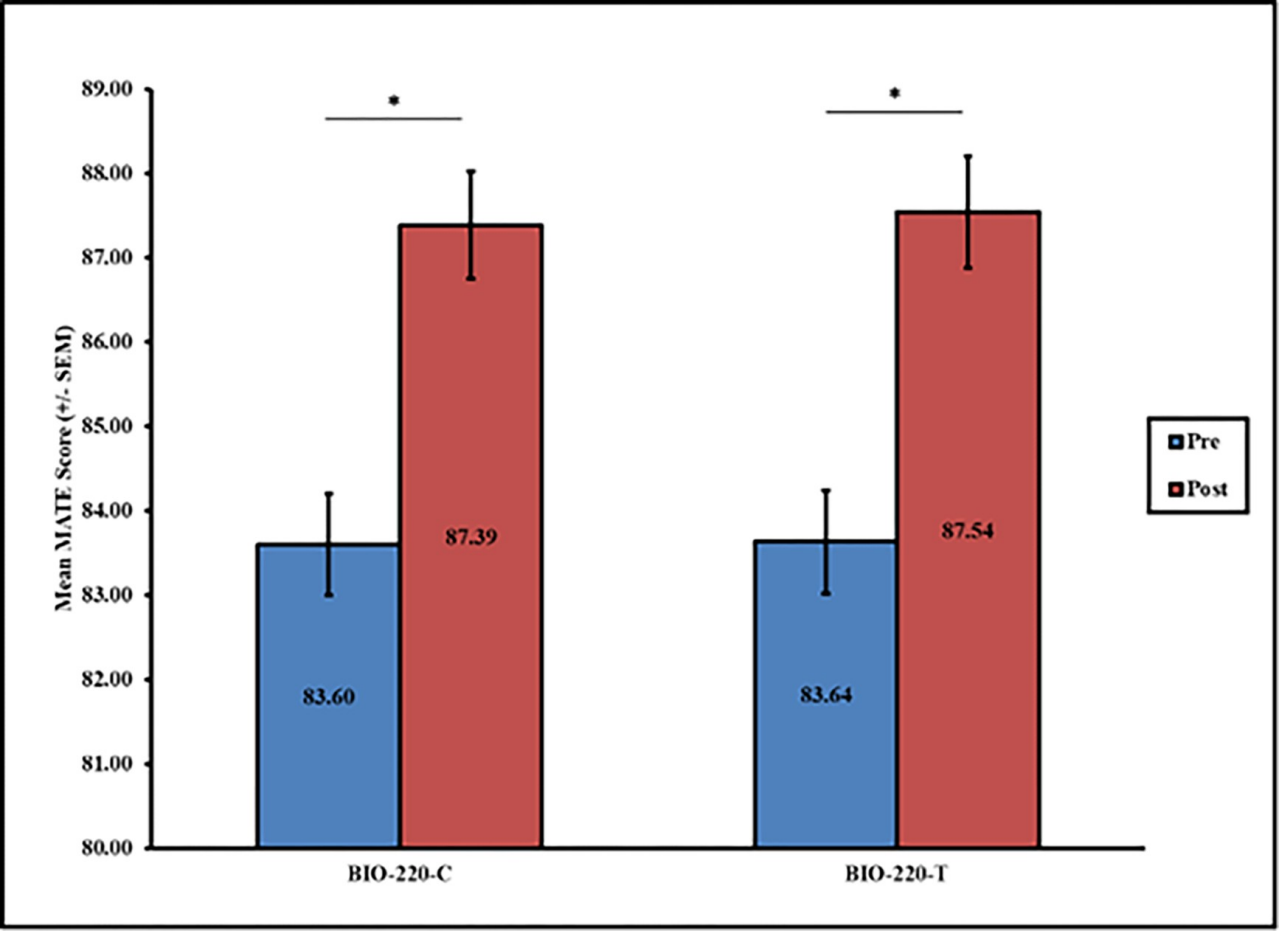
Changes in pre/post acceptance of evolution for BIO 220-T and BIO 220-C. *Note*. * p < 0.05.

Overall participants significantly improved their acceptance of evolution in both BIO 220-T and BIO 220-C pre-to-post (Fig 2). In BIO 220-T, the mean MATE score increased from 83.64 (SD = 11.25) to 87.54 (SD = 11.68, t = -8.50, df = 312 p < 0.001, d = 8.15). In BIO 220-C, the mean MATE score increased from 83.60 (SD = 10.65) to 87.39 (SD = 10.80, t = -8.49, df = 313, p < 0.001, d = 7.90). Perhaps unsurprisingly, participants who entered the course with high acceptance had significantly lower MATE change scores than their counterparts who entered the course with low or medium acceptance across both groups (t = 4.60, df = 625, p < 0.001).

**Fig 3:**
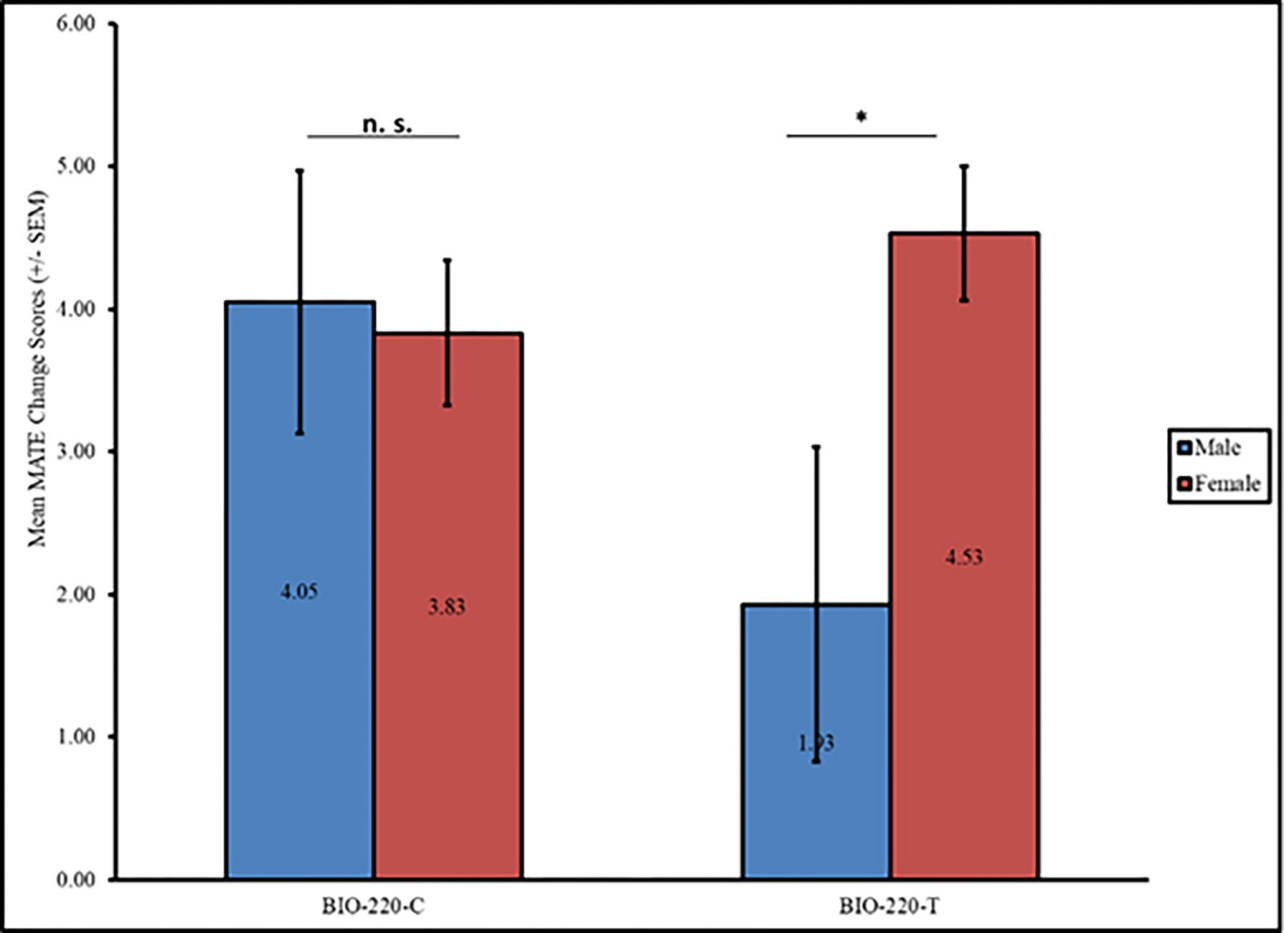
Differences in change in acceptance of evolution by gender for BIO-220-C and BIO-220-T groups. *Note*. * p < 0.05.

When examining participants’ evolution acceptance between BIO 220-T and BIO 220-C, we found no differences on either the pre- or post-scores (p > 0.05 for both analyses). There were also no differences in pre or post acceptance of evolution between BIO-220-T and BIO-220-C for either women or men (p > 0.05). However, when comparing changes in acceptance of evolution for men and women participants in each group, women had significantly larger changes in acceptance (M = 4.54, SD = 7.30) than men (M = 1.93, SD = 10.25, t = -2.42, df = 311, p = 0.016, d = 8.1) in BIO-220-T. Yet no gender differences existed between women (M = 3.83, SD = 7.66) and men (M = 4.05, SD = 8.33, t = 0.22, df = 309, p > 0.05; Fig 3) in BIO 220-C. In other words, it appears there were no differences in evolution acceptance between the groups, but that differences existed between men and women based on whether they were in the treatment or control group, and those differences were practically significant, with a large effect size, for men and women in the treatment group.

Among participants who entered the course with high acceptance, post scores were significantly higher in BIO 220-T (M = 92.04, SD = 8.87) as compared with BIO 220-C (M = 90.38, SD = 8.62, t = -2.08, df = 478, p = 0.038, d = 8.74). There were no such differences in the pre scores of BIO 220-T (M = 88.77, S = 7.41) and BIO 220-C (M = 87.54, SD = 7.25, t = -1.83, df = 478, p > 0.05) for those coming in with high acceptance. In other words, participants who entered the course with high acceptance in BIO 220-T had significantly greater acceptance on the post-survey than their counterparts in BIO 220-C, with a large effect size.

RQ2: *What shifts*, *if any*, *occurred in students’ NOS understanding after explicit*, *reflective instruction on NOS*, *and what differences existed in NOS understanding between students who received the NOS instruction and those who did not*?

Overall, participants’ NOS understanding changed in unpredictable ways over the study (Table 4). There were no significant changes in participants’ understanding that *science is dynamic* for either BIO-220-T or BIO-220-C, yet participants’ understanding that *scientist use creativity* significantly increased for both groups. However, participants’ understanding of *science as subjective* significantly decreased for both BIO-220-T and BIO-220-C, meaning participants viewed science as more objective over time. Finally, participants in BIO-220-T had no changes in their understanding that *science is culturally embedded*, whereas participants’ understanding in BIO-220-C significantly decreased. For all significant differences, effect sizes were small and not practically significant.

**Table 4:** Summary of changes in participants’ NOS understandings.

NOS understanding	BIO-220-T (n = 313)			BIO-220-C (n = 313)		
	Pre Mean (SD)	Post Mean (SD)	Effect size (Cohen’s d)	Pre Mean (SD)	Post Mean (SD)	Effect size (Cohen’s d)
*Science is dynamic*	4.32 (.422)	4.30 (.56)	---	4.26 (.41)	4.22 (.51)	---
*Scientists use creativity*	3.56 (.76)	3.77 (.76)***	.71	3.54 (.72)	3.68 (.80)**	.73
*Science is culturally embedded*	3.75 (.80)	3.66 (.89)	---	3.66 (.82)	3.51 (.94)**	.94
*Science is subjective*	3.28 (.73)	3.14 (.78)**	.78	3.30 (.63)	3.16 (.71)**	.68

*Note*. NOS understanding measured by SUSSI factors reported in Table 2. Likert scale (1 = strongly disagree, 5 = strongly agree).

*p < 0.05

**p < 0.01

***p < 0.001. Effect sizes calculated for significant differences only.

We also examined differences in pre and post NOS understandings and found there were almost no significant differences between the two groups, with two exceptions. Participants in BIO-220-T had a better understanding of science as dynamic than BIO-220-C participants on the pre-survey (p = 0.041) and a better understanding of science as culturally embedded than BIO-220-C participants on the post-survey (p = 0.039). Further, we found no significant differences between men and women participants’ NOS understandings in either BIO-220-T or BIO-220-C for any of the NOS factors.

RQ3: *To what extent did understanding of evolution as a scientific theory differ between students who did and did not receive explicit*, *reflective instruction on NOS*?

Overall participants in BIO 220-T had a better understanding of evolution as a theory following the unit on evolution than participants in BIO 220-C as observed in both their reflective responses and their evolution as a theory exam question. BIO-220-T participants’ coded and scored reflective responses (range = 0–7) were significantly more aligned with an accurate understanding of evolution as a theory (M = 2.14, SD = 1.08) as compared to BIO 220-C participants (M = 1.73, SD = 1.00), U = 9298.5, Z = -3.422, p = 0.001). No differences existed in the misaligned reflective writing scores for treatment and control, which is not surprising given the limited range of possible scores (0–2).

There was also a significant association between participants’ correct response to the evolution as a theory exam question and intervention group, χ^2^(1) = 54.473, p < 0.001, with a moderately strong association, φ = -.426, p < 0.001. Descriptively, only 63.9% of BIO 220-C participants (n = 94 of 147) correctly answered the exam question, while 97.4% of BIO 220-T participants (n = 149 of 153) correctly answered the question.

RQ4: *What relationships existed between acceptance of evolution*, *understanding of NOS*, *and understanding of evolution as a scientific theory for those who did and did not engage with NOS instruction*? *How did these relationships differ based on gender and prior evolution acceptance levels for each group*?

There existed significant moderate positive correlations between participants’ acceptance of evolution, understanding of NOS, and understanding of evolution as a theory for both treatment and control groups (Table 5). While these relationships appear to be similar across BIO 220-T and BIO 220-C, there are some differences worth noting. First, the relationship between BIO 220-T participants’ acceptance of evolution and understanding of NOS as *dynamic* was significantly more pronounced for at the end of the study (r = 0.642) than in the beginning (r = 0.435), z = 2.559, p = 0.005. This relationship was not significantly different for BIO 220-C participants (r = 0.540 and r = 0.445, respectively), z = 1.067, p = 0.143. Second, the relationship between acceptance of evolution and understanding of NOS as *creative* became more pronounced by the end of the study for BIO 220-T participants and less pronounced for BIO 220-C participants. While the strength of relationship within a group was not significant, the difference in the strength of the post-relationships were different between the groups, z = 1.778, p = 0.038. In other words, BIO 220-T participants who better understood NOS as creative tended to have more acceptance of evolution at the end of the study (r = 0.370), while this relationship was much weaker for BIO 220-C participants (r = 0.179). Third, for BIO 220-C participants there existed a moderate negative significant relationship between acceptance of evolution and understanding of evolution as measured by participants’ score on the exam question (r = -0.273). In other words, participants who did not receive NOS instruction tended to get the exam question correct when they had a lower acceptance of evolution at the end of the study. This relationship was not observed for BIO 220-T participants.

**Table 5:** Correlations between participants’ acceptance of evolution, understanding of NOS, and understanding of evolution.

			NOS_dynamic_		NOS_creative_		NOS_social_		NOS_static_		Evolution understanding^a^	
			Pre-	Post-	Pre-	Post-	Pre-	Post-	Pre-	Post-	Aligned	Exam Q
**Acceptance of Evolution**	Pre-MATE	BIO-220-T	.435**	.428*	.213**	.266**	.117*	.086	-.084	-.061	.042	-.012
		BIO-220-C	.445**	.292**	.206**	.135*	.014	-.026	-.016	-.041	.089	-.273**
												
	Post-MATE	BIO-220-T	.411**	.624**	.139**	.370**	.061	.113*	.016	.027	.026	.033
		BIO-220-C	.461**	.540**	.141**	.179**	.054	.037	-.041	-.009	.085	-.280**

*Note*. Aligned = summed score of qualitative responses aligned with understanding of evolution as a theory (range 0–7).

*p < 0.05

**p < 0.01.

^a^ Spearman’s Rho correlational analyses was used as these variables are non-continuous.

When looking at participants whose acceptance of evolution was high (e.g., MATE > 76), there existed differences between BIO 220-T and BIO 220-C participants’ understanding of NOS. In particular, BIO 220-T participants’ understanding of NOS as *creative* improved twice as much (Mean Δ in NOS_creative_ = .26) as BIO-220-C participants [Mean Δ in NOS_creative_ = .12; F(1, 477) = 4.384, p = 0.037]. This significant difference between the groups in understanding of NOS as creative was not present for participants who held low/medium acceptance of evolution at the beginning of the semester. There were also no differences between the groups in changing NOS understanding for the three other factors.

There were no differences between men and women participants’ changes in understanding of NOS in either BIO 220-T or BIO 220-C when their acceptance of evolution was high (e.g., MATE > 76). However, there were differences between female participants’ change in understanding of NOS as *creative*, with BIO 220-T women improving their understanding twice as much (Mean Δ in NOS_creative_ = .27) as BIO 220-C women participants [Mean Δ in NOS_creative_ = .12; F(1, 354) = 4.058, p = 0.045]. There were no differences in mens’ changing understanding of NOS between the two groups, nor were there any other significant differences in changing understanding of other NOS factors for participants based on gender.

## Discussion

In the present study we examined the impact of an explicit, reflective NOS intervention within an introductory biology unit on evolution on students’ acceptance of evolution, understanding of evolution, and understanding of NOS. We utilized a quasi-experimental approach to compare results between the intervention group (i.e., BIO-220-T) and a control group (i.e., BIO-220-C). Overall, the treatment and control groups experienced similar significant improvement in evolution acceptance over the course of the unit with large effect sizes. We also identified differences in outcomes and relationships between the two groups and between subgroups of students (i.e., gender, incoming level of evolution acceptance) that nuance these findings further. To our knowledge, this study is the first to explore the influence of NOS instruction on evolution acceptance in a higher education context and to disaggregate findings by different variables. Below we discuss these findings and situate them in the literature to demonstrate the contribution of this work to the field.

### Factors influencing evolution acceptance

In the present study we found differing levels of evolution acceptance for students in the treatment and control groups based on gender. Specifically, women experienced significantly greater improvement in evolution acceptance than men in the treatment group with large practical effects, but no gender differences existed in the control group. This suggests that the NOS instruction may have had a disproportionate impact on women, which aligns with studies on student performance in STEM undergraduate contexts that demonstrate differences for men and women students [32,33]. Conversely, gender is not generally found to be a significant predictor of evolution acceptance [7,8], so our study extends this work to suggest that gender may be important to acceptance of evolution when a NOS intervention is used.

Recent research has also demonstrated the importance of undergraduate STEM students’ intersectional identities on perceptions and persistence [34,35]. While we did not capture other demographic variables in the present study, our differential gendered findings could potentially be explained by other identities students hold. Alternatively, some research has found girls to be more suggestible than boys [36]. If this pattern holds into adulthood, it could provide an alternate explanation for the different responses to our NOS instruction among women and men when gender has not historically been correlated with evolution acceptance. Differences in suggestibility should not affect the results of a correlational study the same way as one that involves an educational intervention and assessment of its impacts, which may help further explain why this study finds gender to be related to acceptance while others have not. Further research is necessary to determine what types of interventions are likely to foster differing reactions among students with varying intersectional identities (e.g., White man, Black woman), as well as explore methods that may be useful in improving the receptiveness of men to messages about science and evolution.

We also found that there were important differences in the relationships between evolution acceptance, evolution understanding, and NOS understanding for treatment and control groups, which align with previous work investigating these relationships. For example, we found that those who received NOS instruction showed greater understanding of evolution as a theory than those in the control group, and that students with higher acceptance in the treatment group also tended to have a better understanding of NOS (particularly the creativity aspect). These findings are consistent with Kim and Nehm [10] who found significant positive correlations between NOS understanding, understanding of evolution, and acceptance of evolution, as well as with Partin et al. [11], who found understanding and acceptance of evolution to be significant predictors of NOS understanding. What is less clear is the directionality of these relationships. In other words, does improvement in understanding of evolution result in improved acceptance of evolution, or is the opposite true? Further research using structural equation modelling (SEM) is needed as a next step to understanding these relationships.

### Impact of NOS instruction

In the present study, we found that students who received the NOS intervention improved their overall understanding of evolution based on both their reflective writing and exam question responses. When examining the coding scheme for the reflective writing responses (i.e., Table 3) alongside the NOS tenets covered in the intervention there is much overlap. For example, during the second NOS intervention day, Researcher B explicitly defined scientific theory, described the characteristics of theories, and articulated the conflation of scientific theories with ‘lay theories’. These important features of theories were inductively identified as codes demonstrating students’ understanding of evolution as a theory. We also observed that students in the treatment group were more capable of correctly characterizing evolution as a scientific theory as seen in their exam responses. The most common answer among students in the control group was (B): it continues to be revised, which prevents the development of a fixed evolutionary law.

While not a true experimental study, the findings suggest that our explicit and minimally contextualized approach was effective in promoting a more in-depth understanding of evolution for students in the treatment group. This is notable given the short duration of our intervention, of which most NOS interventions run multiple weeks or an entire semester [15,23]. These findings also extend the literature on K-12 and higher education NOS instruction that demonstrates the importance of instruction along a context continuum from improved NOS understandings [16,23] to improved understanding of evolution.

However, our findings were more mixed for changes in NOS understandings; both treatment and control students significantly improved their understanding of science as a creative endeavour and significantly decreased in their understanding of science as subjective. Only students in the control group had a significant decrease in their understanding of science as culturally embedded. However, effect sizes for all of these significant differences were minimal. There may be two explanations for these inconclusive and non-practically significant findings. First, in a review of K-12 NOS literature, Cofre and colleagues [23] found that subjectivity and socio-cultural aspects are two of the most challenging NOS understandings to improve, while creativity is one of the easiest tenets to learn. Our findings align with the findings of the review and may suggest that explicit NOS instruction may not be needed to help students understand that science is creative. The limited need for explicit NOS instruction to improve NOS understanding for certain tenets has also been described in prior higher education contexts [15], and further research is needed to better understand when NOS is necessary, and when it is less necessary, for undergraduate STEM students.

Second, we used the SUSSI as it enabled easy data collection and analysis; however, the majority of NOS studies use a version of the Views of Nature of Science (V-NOS) validated open-ended questionnaire [23]. While we conducted Exploratory Factor Analysis to identify the four factors used in our analysis, we were unable to further strengthen the validity of the factor structure using Confirmatory Factory Analysis because of the limited sample size (n = 627) relative to the number of variables (n = 21) and factors (n = 4). From our EFA, there were eight questions that did not load onto any factor, and Factor 1 (Science is Dynamic) encompassed multiple NOS tenets such as the tentative nature of science, understanding of theories and laws, and multiple methods. The combination of NOS tenets across the empirically identified factors, particularly those tenets included in the NOS intervention, as well as the small variance accounted for by the EFA factor structure that was not confirmed with CFA, may explain the lack of differences between treatment and control groups. Further, there are documented challenges with and the disagreements about measuring NOS understandings [see 37]. Thus, using an unvalidated Likert scale survey instrument to measure NOS understandings may have enabled easy data collection and analyses; however, it may not have accurately or reliability captured students’ NOS understandings. Future research on NOS interventions in an evolutionary context using a different instrument to measure NOS understandings is warranted.

Our results and experiences allow us to provide several recommendations for biology instructors who may wish to embed NOS tenets in their instruction. The activities we designed were all explicit/reflective in nature and ranged from highly contextualized to decontextualized, consistent with what prior work has found to be among the most effective strategies for teaching NOS [12,16,20]. However, the nature of the intervention was limited both in duration (60 minutes total) and scope (covering three NOS tenets), and the results revealed our approach to be semi-effective overall and ineffective for particular student groups. A longer intervention spanning additional NOS tenets may be more likely to induce lasting conceptual change. Our findings were consistent with those presented by Cofre and colleagues [23] who found that the creativity of science is one of the easiest NOS understandings to change, while sociocultural and subjectivity aspects are more difficult. In line with these findings, we suggest pairing teaching of NOS tenets that are more resistant to change with those that may be easier to change to maximize intervention effectiveness. Instructors may also consider utilizing associated course lab time as a place to embed NOS activities–we did not do this in our study, but prior work has found that lab time dedicated to ‘doing’ science is a place that could be effective at improving NOS understandings [15,38].

### Limitations

One limitation of this study is the use of the MATE to measure acceptance of evolution. While it is the most common survey used to measure acceptance among the hundreds of studies that have explored the topic, it has been criticized for assuming participants are Judeo-Christian and including items that are not explicitly related to the theory of evolution [39]. In 2022, after our data were collected, a revised MATE 2.0 was published [39].

The use of the SUSSI can also be considered a limitation in this study. This instrument has not been previously validated, and while we were able identify an interpretable factor structure, the reliability of those factors was not particularly strong–particularly for Factor 4.

Other limitations to this study include the generalizability of our findings to individuals other than college students as well as those in other areas of the country, given the documented relationships between evolution acceptance and age/geography [6]. The participants in our study were also mainly pre-health majors coming from within our state and whose parents were college graduates (see Table 1), which may limit the generalizability of these findings to college students at other institutions with dissimilar populations. Our study also did not collect or analyze complete demographic information, including race, ethnicity, or age, so we were not able to study whether any of these factors influenced our results. The instruction we provided was also relatively limited in nature, given that we focused only on three main tenets of NOS—empiricism, theory vs. law, and observation vs. inference–and for a relatively short duration. Future research could explore the efficacy of instruction based on additional tenets of NOS, including the tentativeness of scientific knowledge, subjectivity and objectivity in science, the role of creativity in science, and the myth of the singular scientific method. This could be accomplished by integrating NOS instruction throughout a semester of a course, rather than a three-class period with a total intervention time of 60 minutes.

## Conclusions

In summary, these findings strongly suggest that acceptance and understanding of evolution and understanding of NOS are related, and that evidence-based efforts to improve students’ NOS understanding through targeted interventions may be effective at improving evolution acceptance—particularly among certain populations, such as women and individuals who already have high acceptance of the theory of evolution. These preliminary findings suggest that targeted efforts to improve NOS understanding can have particular benefits for different student populations, including women and individuals who have already largely embraced the theory of evolution. They also point to the need to explore interventions that can reach more resistant populations, including men and individuals with low evolution acceptance.

## Supporting information

S1 Appendix BIO 220 syllabus. (DOCX)

S2 Appendix NOS instruction description. (DOCX)

S3 Appendix MATE and SUSSI reverse coding. (DOCX)
