# Supplementary material for: Teaching nature of science in introductory biology: Impacts on students’ acceptance of biological evolution
Source: PLoS One. 2023 Aug 10;18(8):e0289680. doi: 10.1371/journal.pone.0289680 (PMC10414625; doi:10.1371/journal.pone.0289680)
Supplement: S1 Appendix — (DOCX) [file pone.0289680.s001.docx]

**S1 Appendix**

**BIO 220 Syllabus**

Note: syllabus has been simplified to omit identifiers and specific policies on grading, absences and other administrative elements of the course.

**Course Description**

Like all organisms, humans are the product of a complex, iterative biological process: evolution. But if only the fittest survive, why are there so many species? And if all life shares an ancestor, what do we have in common with a tunicate (and what’s a tunicate)? This semester we will explore how evolution has led to diversity in organismal form and function, resulting in a myriad of unique species on earth. Using examples from across the tree of life, we will examine how natural selection leads to adaptation in morphology and physiology, ultimately providing organisms with the tools to survive and thrive in a wide range of environments. You will also uncover how processes and structures in your own body reflect the evolutionary history of animals from simple multicellular organisms to complex and highly functioning vertebrates.

The theory of evolution is a foundational concept in biology and will be revisited throughout other courses you take in the discipline. More profoundly, evolution has shaped numerous aspects of our lives, from the reason why humans walk on two legs to how oxygen moves around your body to why antibiotic resistant bacteria are on the rise. We will take a comparative approach to studying evolution, which will situate humans in larger patterns of evolution, but will also give you a glimpse into the incredible diversity of life on our planet. This will guide you to developing the tools to think like a biologist and critically evaluate scientific discoveries through the lens of evolutionary biology.

By the end of this course, you will be able to:

-use your knowledge of the theory of evolution to infer an organism’s responses to environmental change

-explain how diversity is generated via evolutionary processes

-relate differences between taxa to their unique evolutionary histories

-recognize the intrinsic, ecological and economic value of biological diversity

-assess how differences in organismal form and function relate to adaptation

-interpret and critically analyze basic biological data

**What does an average class look like?**

As an introductory course, one focus of BIO 220 is to provide you with foundational knowledge in evolutionary biology. However, this course is not just about learning key concepts, it is also about applying these concepts and interpreting information from other studies, other courses and the world in general. To evaluation your understanding of material and your ability to apply the concepts you are learning in the course, classes will be a mixture of lecture, short peer discussions, and daily in-class questions using iClicker (see below). By working together within the lecture hall to explore ideas and answer questions, you will not only review your knowledge of course content but you will also practice using that knowledge in a friendly, low-stakes environment. In-class experiences will help me support your learning by identifying areas or ideas which we need to revisit or reconsider. iClicker questions, along with homework assignments and post-lab exercises will provide you with preparation for course exams.

**Course Pre-requisites**

There are no course prerequisites. This course serves as a prerequisite for upper-level Biology courses.

**Course Materials**

-Campbell Biology, 11th Edition, with Mastering Biology. Mastering Biology is a graded component of the course (see Mastering Biology information in syllabus) and an access code is packaged with the loose-leaf version of the textbook at the bookstore.

-Custom lab manual, available only through the bookstore.

-Dissecting kits will be required for some labs. We recommend you purchase a dissecting kit from the bookstore.

**-**i>Clicker - not required, but highly recommended. Using iClickers can help students determine whether they are understanding class material in real time and also helps the instructor evaluate whether certain concepts need further explanation. Students who use and properly register an iClicker can earn a bonus on their overall semester score.

**Exams and course grades**

You will take **four** exams (including the final) during the semester. These will be equally weighted and, in total, account for **70%** of your course grade (17.5% each). Lab assignments will account for **20%** of your course grade and homework will account for the remaining **10%**. You may collaborate with classmates on homework and lab assignments unless otherwise indicated.

**Exams:** Exams will assess your comprehension and application of course material. Questions will focus on using knowledge gained from lectures, labs and your textbook to identify evolutionary mechanisms and relationships, assess how structure and function reflect adaptation and compare how different organisms, and organ systems, perform in their associated environments. Exams will assess your understanding of foundational knowledge as well as your ability to apply that knowledge to new examples or scenarios. Exams are not cumulative; however, they will build upon concepts and skills learned in previous units of the course. We will follow up the first two midterm exams with an optional post-exam survey to help you identify areas of strength and areas that require further work.

**Pre- and post-lab assignments:** Labs are an opportunity to gain hands-on experience with the theories, organisms and processes that we discuss in lecture. To prepare you for each lab, you will complete a short pre-lab quiz that will evaluate close reading of the lab’s goals and tasks. Post-lab assignments will assess your understanding of key observations and concepts covered in the lab activity and connect them to lecture content. Pre-lab activities will be completed individually on our LMS, while post-lab activities will usually be completed as a group and handed in at the end of each lab activity. I strongly recommend keeping a copy of all post-lab assignments to help you in preparation for exams. If you have questions or concerns about either pre- or post-lab assignments, I am happy to review your work in the two weeks after your assignment has been returned.

**Homework:** To practice applying your knowledge, you will complete regular homework assignments through Mastering Biology, an online website associated with your textbook (see technical directions below). Homework will help you review course content, explore new examples of key concepts and identify common misconceptions. These assignments will be relatively short and will often reflect the types of questions you will encounter on an exam. You may use your textbook, notes, and work with classmates.

**Participation (Bonus):** We will evaluate comprehension and application of lecture material in real-time using iClickers. During each lecture, I will periodically pose a question that will integrate ideas from past lectures, evaluate your understanding of key ideas or ask you to work through a problem pertaining to the topic at hand. These questions will help me to assess whether we need to revisit or delve deeper into particular topics and will help you to self-assess material where you may need further review.

**Lab Attendance and policies**

Weekly labs provide a deeper and active exploration of key topics, as well as an opportunity to interact and collaborate with your peers. This is an essential component of the course and lab attendance is therefore required at *every* lab section throughout the semester. Unexcused absences will result in a zero for associated lab assignments and violations of lab policies will result in penalties associated with the graded components of that lab. **Students who have missed, or anticipate missing, a lab must contact the lab coordinator, by email as soon as possible.**

**General Course policies**

**Taking exams:** Exams will be given during the Discussion section as indicated in the course schedule. Please mark down these dates in your calendar. If you find that you are unable to take an exam on the scheduled date due to illness, unexpected personal issues or other legitimate reasons, you may be able to re-schedule exams. If you should miss one of the midterm exams, an additional *comprehensive* exam will serve to replace the missing exam score. Exam scores will be reported and recorded on a 100-point scale.

**Grading:** At the end of the course, numerical scores will be compiled, rounded to whole numbers, and translated to letter grades as follows: 97-100 A+, 94-96 A, 90-93 A-, 87-89 B+, 84-86 B, 80-83 B-, 77-79 C+, 74-76 C, 70-73 C-, 67-69 D+, 64-66 D, 60-63 D-, <60 F.

**Collaboration:** You are encouraged to collaborate with classmates on Mastering Biology homework and lab assignments. Because these assignments are an opportunity for you to practice applying your knowledge and to self-evaluate your level of comprehension, you may find it more effective to work in groups. However, copying another student’s work would not only be inappropriate, it would defeat the purpose of enhancing your learning.

**Academic integrity:** Every student in this course is expected to fully comply with all of the provisions of university’s honor code.

**Schedule**

| # | Lecture | Lab |
| --- | --- | --- |
| 1 | INTRODUCTION | Lab 1: Intro and evolution basics |
| 2 | The theory of evolution by means of natural selection |  |
| 3 | Evidence for evolution | No Lab |
| 4 | Genetic variation & the Hardy-Weinberg Equation |  |
| 5 | Mechanisms of evolution |  |
| 6 | Biological species concept & reproductive isolation | Lab 2: Hardy-Weinberg |
| 7 | Speciation & hybridization |  |
| 8 | Origins of life on earth |  |
| 9 | Extinctions, radiations & evolutionary trends | Lab 3: Coevolution |
| 10 | Introduction to phylogenetics |  |
| - | EXAM 1 – NO LECTURE |  |
| 11 | Building a phylogenetic tree | Lab 4: Phylogenetics |
| 12 | Evolutionary time and the tree of life |  |
| 13 | Prokaryotes: bacteria and archaea |  |
| 14 | Protists | Lab 5: Bacteria |
| 15 | Fungi |  |
| 16 | Nonvascular plants |  |
| 17 | Seedless vascular plants & gymnosperms | Lab 6: Protists & Fungi |
| 18 | Angiosperms |  |
| - | EXAM 2 – NO LECTURE |  |
| 19 | Animals: evolutionary history & basic body plans | Lab 7: Plants |
| 20 | Invertebrates I: Sponges, worms and molluscs |  |
| 21 | Invertebrates: Arthropods |  |
| - | SPRING BREAK |  |
| 22 | Early vertebrates, sharks, rays and fish | Lab 8: Invertebrates |
| 23 | Reptiles, birds and amphibians |  |
| 24 | Mammals |  |
| 25 | Human evolution | Lab 9: Diversity and tree-thinking |
| 26 | Introduction to animal form and function |  |
| 27 | Thermoregulation and osmoregulation |  |
| 28 | Excretion | Lab 10: Presentations |
| 29 | Digestion |  |
| - | EXAM 3 – NO LECTURE |  |
| 30 | Reproduction | Lab 11: Digestion & reproduction |
| 31 | Circulation I |  |
| 32 | Circulation II |  |
| 33 | Respiration | Lab 12: Circulation & respiration |
| 34 | Neurons |  |
| 35 | Nervous systems |  |
| 36 | The vertebrate brain | Lab 13: Nervous system |
| 37 | Sensory systems |  |
| 38 | Integration of organ systems |  |
| 39 | SYNTHESIS |  |
| - | EXAM 4 |  |

Readings for Campbell 11^th^ edition by section (for eTextbook)

| # | Date | Lecture | Readings by chapter / section (Campbell 11^th^ etext) | Lab |
| --- | --- | --- | --- | --- |
| 1 | Jan 14 | INTRODUCTION | none | Lab 1: Intro and evolution basics |
| EVOLUTION | | | | |
| 2 | Jan 16 | The theory of evolution by means of natural selection | 22.1, 22.2 |  |
| 3 | Jan 18 | Evidence for evolution | 22.3 | No Lab |
| 4 | Jan 23 | Genetic variation & the Hardy-Weinberg Equation | 23.1, 23.2 |  |
| 5 | Jan 25 | Mechanisms of evolution | 23.3, 23.4 |  |
| 6 | Jan 28 | Biological species concept & reproductive isolation | 24.1, 24.2 | Lab 2: Hardy-Weinberg |
| 7 | Jan 30 | Speciation & hybridization | 24.3, 24.4 |  |
| 8 | Feb 1 | Origins of life on earth | 25.1-25.3 |  |
| 9 | Feb 4 | Extinctions, radiations & evolutionary trends | 25.4-25.6 | Lab 3: Coevolution |
| 10 | Feb 6 | Introduction to phylogenetics | 26.1, 26.2 |  |
| - | Feb 8 | EXAM 1 @ 3pm – NO LECTURE | **Lectures 1-10** |  |
| 11 | Feb 11 | Building a phylogenetic tree | 26.3 | Lab 4: Phylogenetics |
| 12 | Feb 13 | Evolutionary time and the tree of life | 26.4-26.6 |  |
| DIVERSITY | | | | |
| 13 | Feb 15 | Prokaryotes: bacteria and archaea | 27.1-27.3, 27.5, 27.6 |  |
| 14 | Feb 18 | Protists | Ch. 28 | Lab 5: Bacteria |
| 15 | Feb 20 | Fungi | Ch. 31 |  |
| 16 | Feb 22 | Nonvascular plants | 29.1, 29.2 |  |
| 17 | Feb 25 | Seedless vascular plants & gymnosperms | 29.3, 30.1, 30.2 | Lab 6: Protists & Fungi |
| 18 | Feb 27 | Angiosperms | 30.3, 30.4 |  |
| - | Mar 1 | EXAM 2 @ 3pm – NO LECTURE | **Lectures 11-18** |  |
| 19 | Mar 4 | Animals: evolutionary history & basic body plans | Ch. 32 | Lab 7: Plants |
| 20 | Mar 6 | Invertebrates I: Sponges, worms and molluscs | 33.1-33.3 |  |
| 21 | Mar 8 | Invertebrates: Arthropods | 33.4 |  |
| - | Mar 11-15 | SPRING BREAK |  |  |
| 22 | Mar 18 | Early vertebrates, sharks, rays and fish | 34.1-34.3 | Lab 8: Invertebrates |
| 23 | Mar 20 | Reptiles, birds and amphibians | 34.4, 34.5 |  |
| 24 | Mar 22 | Mammals | 34.6 |  |
| 25 | Mar 25 | Human evolution | 34.7; supplementary reading | Lab 9: Diversity and tree-thinking |
| ANIMAL FORM AND FUNCTION | | | | |
| 26 | Mar 27 | Introduction to animal form and function | 40.1, 40.2 |  |
| 27 | Mar 29 | Thermoregulation and osmoregulation | 40.3, 40.4, 44.1 |  |
| 28 | Apr 1 | Excretion | 44.2-44.4 | Lab 10: Presentations |
| 29 | Apr 3 | Digestion | 41.2-41.4 |  |
| - | Apr 5 | EXAM 3 @ 3pm – NO LECTURE | **Lectures 19-28** |  |
| 30 | Apr 8 | Reproduction | 46.1, 46.2, Figure 46.11 | Lab 11: Digestion & reproduction |
| 31 | Apr 10 | Circulation I | 42.1, 42.2 |  |
| 32 | Apr 12 | Circulation II | 42.3, 42.4 |  |
| 33 | Apr 15 | Respiration | 42.5-42.7 | Lab 12: Circulation & respiration |
| 34 | Apr 17 | Neurons | 48.1-48.3 |  |
| 35 | Apr 19 | Nervous systems | 49.1, 49.2 |  |
| 36 | Apr 22 | The vertebrate brain | 49.2-49.5 | Lab 13: Nervous system |
| 37 | Apr 24 | Sensory systems | 50.1-50.4 |  |
| 38 | Apr 26 | Integration of organ systems | Supplementary reading |  |
| 39 | Apr 29 | SYNTHESIS | none |  |
| - | May 6 | EXAM 4 @ 7PM | **Lectures 29-39** |  |
