# Supplementary material for: Teaching nature of science in introductory biology: Impacts on students’ acceptance of biological evolution
Source: PLoS One. 2023 Aug 10;18(8):e0289680. doi: 10.1371/journal.pone.0289680 (PMC10414625; doi:10.1371/journal.pone.0289680)
Supplement: S2 Appendix — (DOCX) [file pone.0289680.s002.docx]

**S2 Appendix**

**NOS Instruction Description**

During three different class periods, Researcher B and C used explicit, reflective instructional methods to teach students about three different NOS tenets: Science is empirical, Theories explain and laws describe, and scientific knowledge is based on both observation and inference.

**NOS Activity 1: Science is empirical and based on evidence**

Given the importance of religiosity in predicting individual’s acceptance of evolution [7, 8], we wanted to start the NOS intervention by addressing that science is just one way of knowing and does not have to be in conflict with other ways of knowing. With this in mind, we used a minimally contextualized activity that started by asking students to use an interactive polling system to provide a few words to answer the question “what is science?” This was then used to create a word cloud that served as the jumping off point for a brief lecture on ‘what is science.’

Students then worked in small groups to brainstorm the goals, evidence, and questions that science, art, and religion seek to answer about the world. After completing the activity, each group electronically submitted their responses. These responses were anonymously shown as Researcher B, who taught the lesson and then went through the differences between science, art, and religion as ways of knowing, emphasizing that science is based upon evidence and seeks to understand the natural world. Researcher B provided an example of how ‘light’ can be understood from scientific, artistic, and religious perspectives and concluded by explaining that a deep understanding of science as a way of knowing includes scientific knowledge, scientific practices, and Nature of Science. The class then transitioned in to a mini-lecture by Researcher C, the primary course instructor, introducing evolutionary theory before ending the class.

**NOS Activity 2: Theories and laws are different scientific knowledge**

Knowing the common misconceptions students hold about theories and laws [23], our goal for this activity was to help students gain a solid understanding of theories and laws before learning about evolution in depth. This moderately contextual activity started by asking students an iClicker question on the relationship between theories and laws. The majority of students (70%) chose the response “Theories are unproven laws; once a theory is proven true, it becomes a law”, indicating they indeed held misconceptions about theories and laws. Next followed a mini-lecture on theories and laws where Researcher B shared an example theory, the genetic theory, and its associated law, the law of independent assortment, with the dates each were developed to illustrate that the theory was developed *after* the law. This process was repeated with one other scientific theory and law, ending with the formal definitions of scientific theories – an evidence-based explanation for observed facts - and laws – a mathematical description of a natural phenomenon.

Next, Researcher B had students engage in a ‘Mystery Tube’ activity (Figure 1) [16]. Students observed pulling of the mystery tube strings and directed Researcher B in pulling the different strings. Students were then tasked with working in small groups to develop a ‘Law of strings’ and ‘String theory’ to describe and explain the observed string movement, respectively. Students were able to borrow and manipulate one of the four mystery tubes rotating around the classroom and were encouraged to draw out their string theory. Researcher B then displayed student’s different string theory drawings on the document camera to illustrate that there are different ways to explain the law of strings. Researcher B concluded the activity by explaining why scientific theories are continually conflated with lay-theory and posed the same iClicker question at the end, where 98% of students correctly chose “Laws describe and theories explain scientific phenomenon”. Researcher C then transitioned into a brief history of evolution before moving on to describing natural selection. Following the class, students were instructed to watch a short video on the theory evolution that focused on the scientific definition of theory.


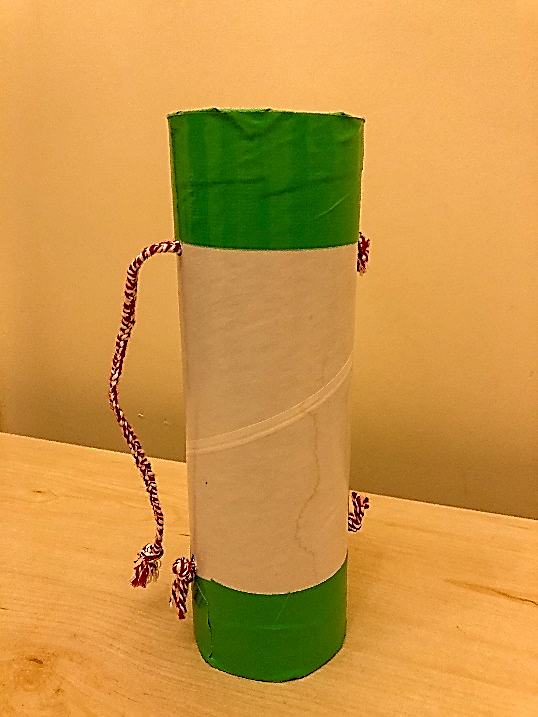


**Fig 1. Mystery tube used to help students apply their understanding of theories and laws.**

**NOS Activity 3: Scientific knowledge requires both observation and inference**

The third activity addressed the NOS tenet that scientific knowledge is based on both observation *and* inference and used a highly contextualized activity to help students reflect on how these concepts differ. Researcher C presented students with drawings of two fish of the same species (threespine sticklebacks, *Gasteroseus aculeatus*) found in different environments. In this particular species, several physical aspects vary depending on their environment. In groups, students were tasked with examining these images, first recording observations about the two fish images, then what they could infer about the fish. Students shared their observations and inferences using an interactive polling system. Researcher C then provided specific definitions of the two terms, highlighting how observations and inferences differ and reviewed the students’ responses. In small groups, students identified observations and/or inferences that were miscategorized and discussed how these two types of information help biologists draw conclusions about the natural world. After the activity, Researcher C used these same fish as an example of evolution, explaining how the observed differences helped researchers infer that differences in the traits of these fish reflect adaptations to different environments.
