# Supplementary material for: Teaching nature of science in introductory biology: Impacts on students’ acceptance of biological evolution
Source: PLoS One. 2023 Aug 10;18(8):e0289680. doi: 10.1371/journal.pone.0289680 (PMC10414625; doi:10.1371/journal.pone.0289680)
Supplement: S3 Appendix — (DOCX) [file pone.0289680.s003.docx]

**S3 Appendix**

**MATE and SUSSI Reverse Coding**

**Measure of Acceptance of the Theory of Evolution (MATE)^[[1]](#footnote-1)^**

1=strongly disagree, 2=disagree, 3=undecided, 4=agree, 5=strongly agree

Organisms existing today are the result of evolutionary processes that have occurred over millions of years.

The theory of evolution is incapable of being scientifically tested. (reverse coded)

Modern humans are the product of evolutionary processes that have occurred over millions of years.

The theory of evolution is based on speculation and not valid scientific observation and testing. (reverse coded)

Most scientists accept evolutionary theory to be a scientifically valid theory.

The available data are ambiguous (unclear) as to whether evolution actually occurs. (reverse coded)

The age of the earth is less than 20,000 years. (reverse coded)

There is a significant body of data that supports evolutionary theory.

Organisms exist today in essentially the same form in which they always have. (reverse coded)

Evolution in not a scientifically valid theory. (reverse coded)

The age of the earth is at least 4 billion years.

Current evolutionary theory is the result of sound scientific research and methodology.

Evolutionary theory generates testable predictions with respect to the characteristics of life.

The theory of evolution cannot be correct since it disagrees with the Biblical account of creation. (reverse coded)

Humans exist today in essentially the same form in which they always have. (reverse coded)

Evolutionary theory is supported by factual historical and laboratory data.

Much of the scientific community doubts if evolution occurs. (reverse coded)

The theory of evolution brings meaning to the diverse characteristics and behaviors observed in living forms.

With few exceptions, organisms on earth came into existence at about the same time. (reverse coded)

Evolution is a scientifically valid theory.

**Student Understanding of Science and Scientific Inquiry (SUSSI)^[[2]](#footnote-2)^**

1=strongly disagree, 2=disagree, 3=undecided, 4=agree, 5=strongly agree

*Factor 1: Science is dynamic*

Scientific theories may be completely replaced by new theories in light of new evidence.

Scientific theories may be changed because scientists reinterpret existing observations.

Scientific theories are subject to on-going testing and revision.

Scientists may make different interpretations based on the same observations.

Scientists use a variety of methods to produce fruitful results

Experiments are not the only means used in the development of scientific knowledge.

*Factor 2: Scientists use creativity*

Scientists do not use their imagination and creativity because these conflict with their logical reasoning. (reverse coded)

Scientists do not use their imagination and creativity because these can interfere with objectivity. (reverse coded)

Scientists use their imagination and creativity when they analyze and interpret data.

*Factor 3: Science is culturally embedded*

Cultural values and expectations determine what science is conducted and accepted

Cultural values and expectations determine how science is conducted and accepted.

*Factor 4: Science is subjective*

When scientists use the scientific method correctly, their results are true and accurate. (reverse coded)

Scientists observations of the same event will be the same because observations are facts (reverse coded)

Scientists follow the same step-by-step scientific method. (reverse coded)

*Did not load onto a factor*

Scientists observations of the same event may be different because the scientists’ prior knowledge may affect their observations.

Unlike theories, scientific laws are not subject to change. (reverse coded)

Scientific theories based on accurate experimentation will not be changed. (reverse coded)

Scientific theories exist in the natural world and are uncovered through scientific investigations.

Scientific laws are theories that have been proven. (reverse coded)

Scientists observations of the same event will be the same because scientists are objective. (reverse coded)

All cultures conduct scientific research the same way because science is universal and dependent of society and culture. (reverse coded)

1. Rutledge, M., and Warden, M. (1999). *School Science and Mathematics, 99*(1): 13-18. [↑](#footnote-ref-1)
2. Liang, L., Chen, S., Chen, X., Kaya, O., Adams, A., Macklin, M., and Ebenezer, J. (2008). Preservice teachers’ views about nature of scientific knowledge development: An international collaborative study. *International Journal of Science and Mathematics Education, 7*: 987-1012. [↑](#footnote-ref-2)
